# All-trans retinoic acid modulates proliferation and apoptosis of secondary hair follicle–dermal papilla cells in cashmere goats via the TGF-*β*2/Smad2/3 pathway

**DOI:** 10.3389/fvets.2026.1765302

**Published:** 2026-03-18

**Authors:** Yadong Gao, Guizhen Gao, Xiao Zhang, Wei Lian, Xueyong Wu, Kun Cui, Lei Zhu, Guoqing Jiang, Ziyang Xu, Jiawei Wang, Mingli Peng, Rui Ding, Dongjun Liu, Ming Cang, Fei Hao

**Affiliations:** 1 Key Laboratory of Reproductive Regulation and Breeding of Grassland Livestock, School of Life Sciences, Inner Mongolia University, Hohhot, China; 2 Peking University Cancer Hospital (Inner Mongolia Campus), Affiliated Cancer Hospital of Inner Mongolia Medical University, Hohhot, China

**Keywords:** all-trans retinoic acid (ATRA), cell apoptosis, cell proliferation, secondary hair follicle–derived dermal papilla cells (SHF-DPCs), TGF-*β*2/Smad2/3 pathway, transcriptome sequencing

## Abstract

Cashmere, a fiber of high economic value, is produced by secondary hair follicles (SHFs), whose growth depends on the proliferation and apoptosis of SHF-derived dermal papilla cells (SHF-DPCs). All-trans retinoic acid (ATRA), a metabolite of vitamin A, has shown inconsistent effects on hair follicle biology, with reports of both inhibition and stimulation. To clarify the role of ATRA in follicular development, we investigated its effects on the proliferation and apoptosis of *in vitro*–cultured SHF-DPCs isolated from *Capra hircus* (Albas cashmere goats). SHF-DPCs were obtained from scapular skin, and the optimal ATRA concentration (10^−4^ M) and treatment duration (24 h) were identified using Cell Counting Kit-8, 5-ethynyl-2′-deoxyuridine, and terminal deoxynucleotidyl transferase dUTP nick end labeling assays. Cells were then assigned to an ATRA-treated group (10^−4^ M) or a control group. Proliferation, apoptosis, and cell cycle progression were evaluated, followed by transcriptome sequencing. Transcriptomic analysis indicated enrichment of the transforming growth factor-*β* (TGF-*β*) signaling pathway. Therefore, mRNA and protein levels of TGF-*β*2—a follicle growth–related regulator—and Smad2/3 phosphorylation were examined. The TGF-*β* type I/II receptor inhibitor LY2109761 was used to further validate the involvement of this pathway. ATRA inhibited SHF-DPC proliferation by inducing G1-phase cell cycle arrest and promoted apoptosis by upregulating Bax expression while downregulating Bcl-2 protein expression. Transcriptomic analysis showed that ATRA upregulated *TGFβ2* expression in SHF-DPCs. Gene Ontology analysis revealed enrichment of genes related to proliferation and apoptosis, such as “cell migration” and “cell cycle.” Kyoto Encyclopedia of Genes and Genomes pathway analysis identified the TGF-*β* signaling pathway as a major regulatory target. Treatment with LY2109761 partially reversed ATRA-induced reductions in proliferation, increases in apoptosis, and elevations in Smad2/3 phosphorylation. Overall, ATRA inhibits proliferation and promotes apoptosis in SHF-DPCs, likely through the TGF-*β*2/Smad2/3 pathway. These findings provide novel insights into ATRA-mediated regulation of hair growth and offer a theoretical basis for future research on cashmere goat SHF development.

## 1 Introduction

As a defining feature of mammals, hair serves essential physiological functions in body protection and thermoregulation. Hair is produced by hair follicles—microscopic appendages in the skin—that possess a distinct anatomical structure consisting of the hair bulb, upper hair bulb, isthmus, and infundibulum (1, 2). Hair follicle formation is driven by reciprocal interactions between the epidermis and dermis, accompanied by cellular proliferation and differentiation, and proceeds through three stages: induction, morphogenesis, and differentiation (3). After formation, hair follicles undergo cyclic growth characterized by alternating phases of anagen (growth), catagen (regression), and telogen (resting) (4, 5). As the foundation of animal pelage, hair follicles are the primary organs responsible for determining hair regenerative capacity (6, 7). The Inner Mongolia Albas cashmere goat, a dual-purpose breed valued for both cashmere and meat, is globally recognized for producing cashmere of exceptional softness, fineness, thermal insulation, and luster—qualities that confer high economic value (8, 9). Cashmere goat hair follicles are classified into primary hair follicles (PHFs) and secondary hair follicles (SHFs) based on their morphogenetic timing and structural features. PHFs generate wool (medullated fibers), whereas SHFs produce cashmere (non-medullated fibers) (10). Therefore, the growth and development of SHFs directly determine cashmere quality and yield.

Dermal papilla cells (DPCs), located at the base of the hair follicle bulb, function as the “command center” of follicular growth. Through the secretion of signaling molecules, DPCs regulate the proliferation and differentiation of hair matrix cells, thereby playing a pivotal role in controlling the hair follicle cycle and maintaining hair growth (11, 12). As targets of small regulatory molecules such as hormones and neuropeptides, DPCs indirectly mediate the effects of these factors on the hair follicle cycle (13). Furthermore, DPCs highly express the anti-apoptotic factor Bcl-2 during telogen and the telogen-to-anagen transition, facilitating the shift from telogen to anagen (14). The critical role of DPCs in hair follicle growth has attracted substantial research interest aimed at elucidating the mechanisms underlying hair growth. The successful *in vitro* culture of DPCs from multiple species has provided a robust model for investigating hair growth regulation and for exploring novel therapeutic approaches to hair-related disorders.

Hair follicle growth and development are precisely regulated by multiple molecular signaling pathways and genes. Studies have shown that key pathways, including Wnt, transforming growth factor-*β* (TGF-β), bone morphogenetic protein (BMP), and Hippo, play essential roles in follicular development. The Wnt pathway, regulated by multiple Wnt ligands, promotes the proliferation and differentiation of hair follicle stem cells via *β*-catenin–mediated gene regulation and interacts with DPC-derived signals to control follicle morphogenesis, cyclic growth, and regeneration (15). The BMP pathway is critical for maintaining DPC function and mediating epithelial–mesenchymal interactions during follicle morphogenesis (16). The Hippo pathway, which is highly conserved in mammals, regulates organ size by controlling cell proliferation, apoptosis, and stem cell self-renewal (17). The TGF-*β* pathway maintains epidermal and hair follicle cycle homeostasis by regulating stem cell proliferation, differentiation, and apoptosis. Disruption of TGF-*β* signaling leads to abnormal proliferation and differentiation of isthmus stem cells, epidermal cells, and sebaceous gland cells; impedes the initiation of the next anagen phase; and results in incomplete hair shaft development (18). TGF-*β*2, a subtype of the TGF-*β* family, exhibits diverse biological functions, including the regulation of cell proliferation, promotion of endothelial fibrosis, and modulation of apoptosis (19–21). Emerging evidence highlights its critical role in hair follicle growth: dihydrotestosterone (DHT) inhibits the proliferation of human keratinocytes and DPCs by upregulating TGF-*β*2 expression, thereby suppressing hair growth (22). Transcriptomic analyses of cashmere goat skin have shown that the catagen and telogen phases are characterized by activation of TGF-*β* signaling together with suppression of cell cycle related pathways, indicating that inhibition of cell proliferation is a hallmark of hair follicle regression (23). Additionally, TGF-*β*2 enhances the phosphorylation of Smad2/3, contributing to DPC apoptosis (18).

Vitamin A, a fat-soluble micronutrient, is essential for hair growth. Its primary bioactive form in animals is all-trans retinoic acid (ATRA), a lipophilic, unsaturated monohydric alcohol (24). ATRA is indispensable for maintaining epithelial integrity, normal vision, reproduction, gene regulation, and antioxidant defense in animals (25–28). In recent years, increasing attention has been directed toward the adverse effects of excessive ATRA on hair follicle growth and development. For instance, ATRA overuse accelerates the transition from anagen to catagen—a process closely associated with hair loss mechanisms (29–31). However, conflicting findings exist: *in vitro* studies have demonstrated that combining ATRA with minoxidil promotes hair growth, a strategy that has been applied in clinical hair loss treatment (32–34). Moreover, ATRA can activate the TGF-*β*/Smad signaling pathway and modulate cell differentiation, proliferation, and apoptosis, suggesting that DPCs may serve as targets of ATRA within hair follicles (35–37). Nevertheless, the effects of ATRA on cashmere goat hair follicle growth and its underlying regulatory mechanisms remain poorly understood. To address this knowledge gap, we isolated and cultured SHF-DPCs from cashmere goats during the anagen phase, optimized the ATRA treatment concentration, and performed RNA sequencing (RNA-seq) analysis. Our results demonstrated that ATRA inhibits SHF-DPC proliferation via the TGF-*β*2/Smad2/3 signaling pathway. These findings elucidate the inhibitory role of ATRA in hair follicle growth, reveal a novel regulatory mechanism, and provide a theoretical foundation for further research on cashmere goat hair follicle development.

## 2 Materials and methods

### 2.1 Ethics statement

All animal experiments were conducted in strict accordance with the Guidelines for the Management and Use of Laboratory Animals established by the Laboratory Animal Research Center of Inner Mongolia University (SYXK2020-0006). The animal experiments in this study were approved and authorized by the Bioethics Committee of Inner Mongolia University (IMU-2025-GOAT-042).

### 2.2 Sample collection and cell isolation

Albas cashmere goats used in this study were provided by Erwei White Cashmere Goat Co., Ltd. (Ordos, Inner Mongolia, China). Healthy adult goats (3 years old, *n* = 6) were randomly selected, and skin samples were collected from the right scapular region during the cashmere anagen phase (September). SHF-DPC isolation and culture followed previously established protocols (38). Briefly, immediately after collection, skin tissues were rinsed three times with sterile phosphate-buffered saline (PBS; Biological Industries, Kibbutz Beit Haemek, Israel). Excess adipose tissue was removed using a sterile scalpel, and the remaining tissue was digested in PBS containing 0.25% neutral protease (Sigma-Aldrich, St. Louis, MO, United States) at 37 °C for 30 min. Post-digestion, tissues were transferred to PBS, and PHFs were separated from SHFs using sterile forceps under a stereomicroscope. Isolated SHFs were digested with type IV collagenase (Sangon Biotech, Shanghai, China), and digestion was terminated with Dulbecco’s modified Eagle medium (DMEM)/F-12 (Biological Industries) supplemented with 10% fetal bovine serum (FBS; Viva Cell, Shanghai, China) (38). SHF-DPCs were cultured in DMEM/F-12 containing 10% FBS at 37 °C in a humidified incubator with 5% CO₂ (39). The medium was refreshed every 2 days, and SHF-DPCs from passages 4–7 were used for subsequent experiments.

### 2.3 Cell Counting Kit-8 (CCK-8) assay for cell viability

Cell viability was assessed using the CCK-8 assay based on the WST-8 reagent (Biosharp, Hefei, China). SHF-DPCs were seeded into 96-well plates at a density of 5 × 10^3^ cells/well in 100 μL of medium and cultured at 37 °C with 5% CO₂ for 24 h. After removal of the complete culture medium, cells were washed with PBS and treated with ATRA (HY-14649, MedChemExpress, Monmouth Junction, NJ, United States) at different concentrations (10^−8^, 10^−7^, 10^−6^, 10^−5^, 10^−4^, 10^−3^ M) for 24, 48 or 72 h. For mechanistic validation experiments, SHF-DPCs were treated with ATRA (10^−4^ M) and/or LY2109761 (2 μM), a TGF-*β* type I/II receptor inhibitor (S2704; Selleck Chemicals, Houston, TX, United States), for 24 h. ATRA and LY2109761 were prepared in complete culture medium, and the control group received an equal volume of culture medium. Following treatment, the existing medium was completely removed, cells were rinsed with PBS, and 100 μL of fresh medium containing 10% CCK-8 reagent was added to each well. After 2 h of incubation, absorbance at 450 nm was measured using a microplate reader (BioTek, Winooski, VT, United States). Each experimental group included seven replicates.

### 2.4 5-Ethynyl-2′-deoxyuridine (EdU) assay for cell proliferation

The EdU assay (KTA2031, Abbkine, Wuhan, China) was used to directly measure DNA synthesis, the most accurate method for assessing cell proliferation. Treated SHF-DPCs were seeded into 24-well plates containing coverslips. Following the manufacturer’s instructions, cells were incubated with the EdU working solution at 37 °C for 2 h, fixed with 4% paraformaldehyde, permeabilized with PBS containing 0.5% Triton X-100 (Sigma-Aldrich), and labeled with the Click-iT reaction mixture. Three independent replicates were performed per group. Fluorescent images were acquired using a laser confocal microscope (Nikon, Tokyo, Japan), and the proportion of EdU-positive cells was quantified using ImageJ software.

### 2.5 Terminal deoxynucleotidyl transferase–mediated dUTP nick end labeling (TUNEL) assay for cell apoptosis

Cell apoptosis was detected using the TUNEL assay (KTA2010, Abbkine). Following the same pre-treatment steps as in the EdU assay, cells were incubated with 500 μL of PBS containing 0.3% Triton X-100 at room temperature for 5 min, rinsed three times with PBS, and incubated with 100 μL of the TUNEL detection solution at 37 °C for 1 h in the dark. After washing, nuclei were stained with DAPI (#10236276001, Sigma-Aldrich). Apoptotic cells were visualized and quantified using a microscope.

### 2.6 Cell cycle analysis

Cell cycle progression was analyzed using a Cell Cycle Assay Kit (c001, 7Sea Biotech, Shanghai, China) and a flow cytometer (FACSAria SORP, BD Biosciences, Franklin Lakes, NJ, United States). Cells were harvested by trypsinization, fixed with ice-cold 70% ethanol for 2 h, centrifuged, and resuspended in propidium iodide (PI) staining solution. After incubation at 37 °C for 30 min in the dark, samples were analyzed via flow cytometry. Three independent replicates were performed per group.

### 2.7 Immunofluorescence staining

Immunofluorescence staining was used to detect the specific expression of *α*-smooth muscle actin (*α*-SMA) and CD133 in cashmere goat SHF-DPCs (38, 40). Briefly, SHF-DPCs were seeded into 24-well plates with coverslips (Shanghai Fan Jing Biological Science and Technology, Shanghai, China) at a density of 3 × 10^5^ cells/well. When cell confluency reached 70%, cells were fixed with 4% paraformaldehyde for 20 min, rinsed three times with PBS, and permeabilized with 0.1% (v/v) Triton X-100 at room temperature for 30 min. Non-specific binding was blocked with 1% bovine serum albumin (BSA) for 30 min. Cells were then incubated overnight at 4 °C with primary antibodies against *α*-SMA (1:200; YT5053; Immunoway, Plano, TX, United States) and CD133 (1:200; YT5192; Immunoway). After washing, cells were incubated with horseradish peroxidase (HRP)–conjugated goat anti-rabbit secondary antibody (1:500; ab150077; Abcam, Cambridge, United Kingdom) at room temperature for 1 h, followed by nuclear staining with DAPI. Fluorescent images were captured and quantified using a confocal microscope.

### 2.8 Quantitative real-time PCR (qRT-PCR)

Total RNA was extracted from cells using RNAiso reagent (Takara Bio Inc., Shiga, Japan). RNA concentration was measured using a NanoDrop Spectrophotometer (Thermo Fisher Scientific, Waltham, MA, United States). cDNA was synthesized using a cDNA Synthesis Kit (Takara Bio Inc.). qRT-PCR was performed on a CFX96 Real-Time PCR System (Bio-Rad Laboratories, Hercules, CA, United States) using the TB Green® Premix Ex Taq™ II (Takara Bio Inc.). The 2^−ΔΔCt^ method was used to calculate relative mRNA expression levels, with *GAPDH* as the internal reference gene. Primer sequences for target genes are listed in Table 1.

**Table 1:** Primer sequences used in this study.

Gene name	Forward primer (5′—3′)	Reverse primer (5′—3′)
*GADPH*	GGTCGGAGTGAACGGAT	TCTGCCTTGACTGTGCC
*TGFβ2*	TTCTGTGGGTACCTTGATGCC	CTCTGGCTTTCGGGTTCTGT
*PCNA*	CTTGAAGAAAGTGCTGGAG	TGGACATGCTGGTGAGG
*Ki67*	AAGAGGCAGCCCAAGTCATC	TTCTTAGCCTACTCCGGCCT
*ATF3*	CCGAGTGGATACAGGAGCAAAA	TGTTCTGGATGGCGAACCTC
*BRD2*	CCCTGAGAGGGGGTTCTTCA	TTCGTAGCTCCTCCATTGGC
*CNTD1*	GCTGTTGCCACAGAGACCAT	CAGGAGAAAAACAAACTCCACGAT
*DDIT3*	ATCGAGGTCAGAGGCTTGAGT	TTCCTTCTGGAACACTCTCTCC
*EFCAB2*	GCAACAAGATGGCGGACGA	GGATAATTGTTCCGACCTCTCTCA
*ATXN10*	GATCGCGAGCACTTTTGTGG	TCAGGCGCAGAAGATCAATCA
*BCL2*	CATGTGTGTGGAGAGCGTCA	CGTGTTTTGATTTCCCAGCCT
*CLSPN*	TGTTGGATGCAGATGGGTTCT	TGCAAACTTCCCGGTACACA
*DMPK*	ACCAGAACTTCGCCAGTCAA	GGCCATATGGGAAGGTGGAT
*ELK3*	GACGCCTGTCAGCATGGAAA	TTGCACTCTCCATACCCAGTT

### 2.9 Protein extraction and Western blotting

Total protein was extracted from SHF-DPCs using a Protein Extraction Kit (CWBIO, Beijing, China), and protein concentration was determined using a BCA Protein Assay Kit (Thermo Fisher Scientific). Equal amounts of protein (10–20 μg per sample) were separated via SDS-PAGE and transferred to methanol-activated PVDF membranes. Membranes were blocked with 5% non-fat milk for 1 h at room temperature, then incubated overnight at 4 °C with the appropriate primary antibody. The following antibodies were used: rabbit monoclonal anti-Bcl-2 (1:1,000; #3498; Cell Signaling Technology, Danvers, MA, United States), rabbit polyclonal anti-Bax (1:5,000; 50,599-2-Ig; Proteintech, Rosemont, IL, United States), rabbit polyclonal anti-TGF-*β*2 (1:1,000; 19,999-1-AP; Proteintech), rabbit monoclonal anti-phospho-Smad2 (1:1,000; #18338; Cell Signaling Technology), rabbit polyclonal anti-Smad2 (1:2,000; 12,570-1-AP; Proteintech), rabbit polyclonal anti-Smad3 (1:1,000; 30,130-1-AP; Proteintech), rabbit polyclonal anti-phospho-Smad3 (1:1,000; YP0363; Immunoway), and rabbit polyclonal anti-GAPDH (1:5,000; 10,494-1-AP; Proteintech).

After washing with TBST, membranes were incubated with HRP-conjugated goat anti-rabbit secondary antibody (1:1,000; SA00001-2; Proteintech) for 1 h at room temperature. Protein bands were visualized using enhanced chemiluminescence reagent (Abbkine) and a Tanon 5,200 imaging system (Tanon, Shanghai, China). Band intensities were quantified using ImageJ software.

### 2.10 Transcriptome sequencing and bioinformatics analysis

SHF-DPCs isolated from three independent individual animals were subjected to drug treatment (*n* = 3). Total RNA was extracted from SHF-DPCs using TRIzol reagent, and genomic DNA was digested with DNase. mRNA was enriched using oligo(dT)-conjugated magnetic beads, followed by library construction. Library quality was validated using an Agilent 2,100 Bioanalyzer (Agilent Technologies, Santa Clara, CA, United States) before sequencing. Raw sequencing reads were processed using fastp software for quality control; low-quality bases (*Q* < 20), sequences containing ≥ 10% N, fragments shorter than 50 bp, and adapter contamination were removed using TrimGalore (v0.6.6) to generate high-quality clean reads for subsequent analysis.

Differential expression analysis was performed using the t.test function in R, with thresholds set as |log₂ fold change (FC)| > 1 and *p* < 0.05 for significantly upregulated or downregulated genes. Hierarchical clustering heatmaps of differentially expressed genes (DEGs) were generated using the pheatmap package in R. Functional annotation of DEGs was conducted using the Gene Ontology (GO) database,^1^ and Kyoto Encyclopedia of Genes and Genomes (KEGG) pathway enrichment analysis was performed using ClusterProfiler.

### 2.11 Statistical analysis

All data are presented as the mean ± standard deviation (SD) and were analyzed using GraphPad Prism 10 software. Differences between two groups were assessed using two-tailed *t*-tests, while one-way analysis of variance (ANOVA) was used for comparisons among three or more groups. Statistical significance was defined as *p* < 0.05, and highly significant differences as *p* < 0.01.

## 3 Results

### 3.1 *In vitro* isolation, identification, and culture of SHF-DPCs

**Figure 1:**
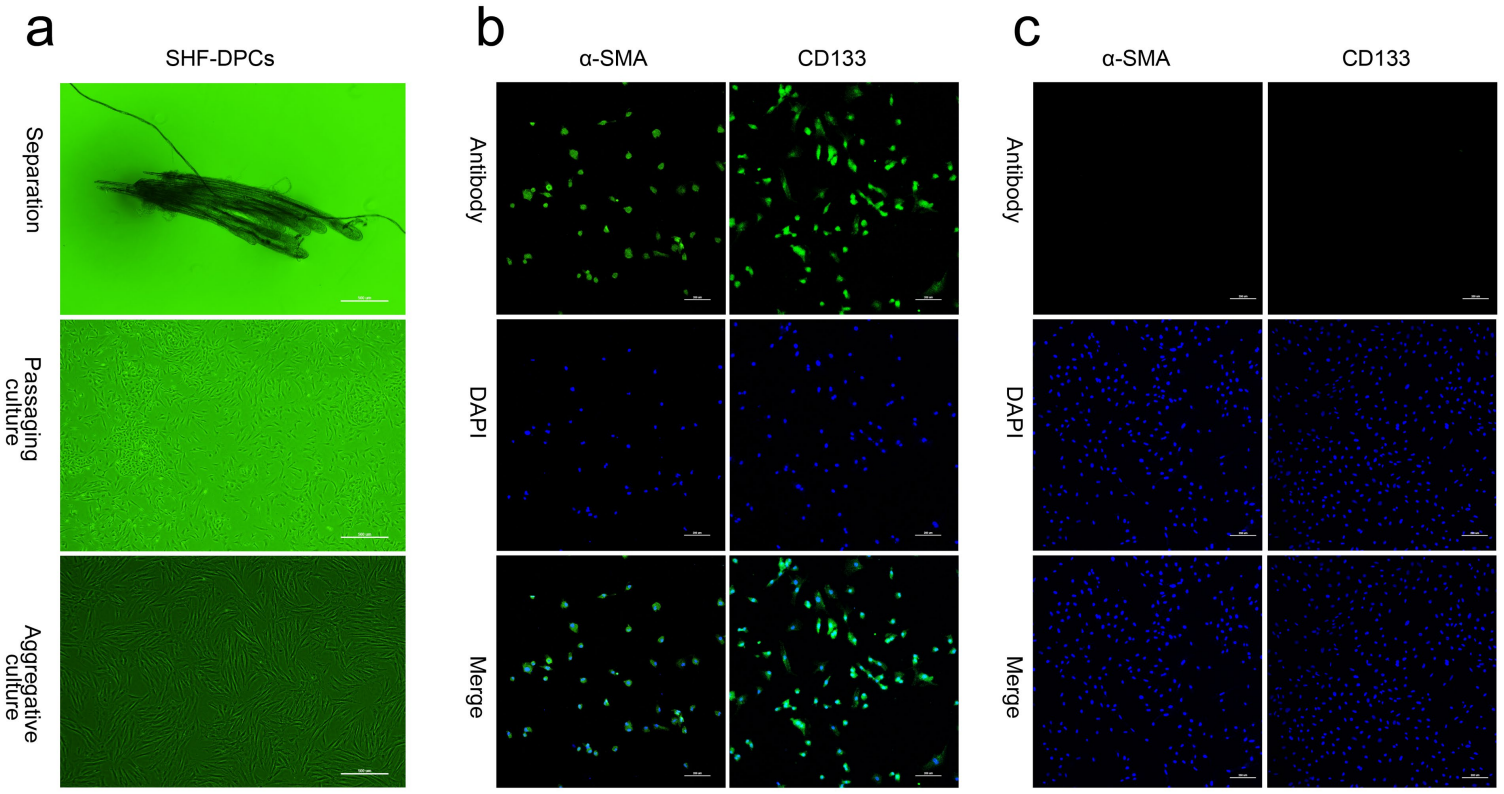
*In vitro* culture and identification of secondary hair follicle–derived dermal papilla cells (SHF-DPCs) from Albas cashmere goats. (**a**) Representative micrographs of isolated SHFs from skin tissue, SHF-DPCs during culture, and purified SHF-DPCs (scale bar = 500 μm; *n* = 3). (**b**) Representative immunofluorescence images of SHF-DPCs stained with the specific markers *α*-smooth muscle actin (*α*-SMA) and CD133 (scale bar = 200 μm; *n* = 3). (**c**) Representative immunofluorescence images of adipose stem cells (negative control) stained with *α*-SMA and CD133 (scale bar = 200 μm; *n* = 3).

SHF clusters were observed surrounding individual thick PHFs in isolated skin tissue. After removal of PHFs, SHFs were collected, and SHF-DPCs migrated out of the follicles within 10 d. Confluent SHF-DPCs exhibited aggregative growth, and purified SHF-DPCs were obtained through gradient digestion (Figure 1a). Immunofluorescence staining using the DPC-specific markers *α*-SMA and CD133 confirmed positive expression in purified SHF-DPCs (Figure 1b), whereas adipose stem cells (negative control) showed no detectable staining (Figure 1c). These findings confirm the successful isolation and culture of SHF-DPCs from Albas cashmere goats.

### 3.2 Effects of ATRA on the proliferation and apoptosis of SHF-DPCs

**Figure 2:**
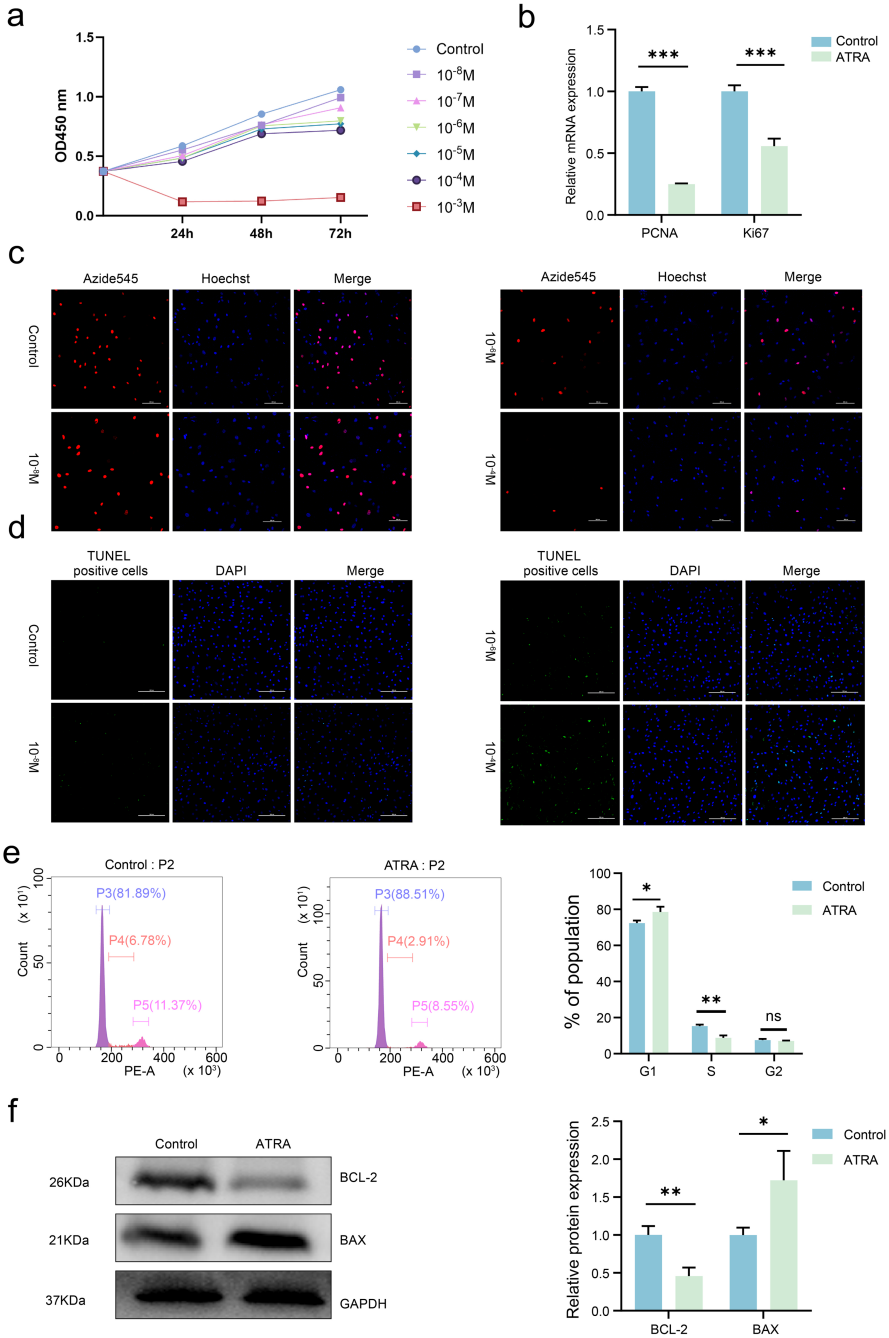
All-trans retinoic acid (ATRA) inhibits proliferation and promotes apoptosis in cashmere goat SHF-DPCs. **(a)** The effects of different concentrations of ATRA (0, 10^−8^, 10^−7^, 10^−6^, 10^−5^, and 10^−4^ M) on the viability of SHF-DPCs were evaluated by the CCK-8 assay after 24, 48, and 72 h of treatment. **(b)** Quantitative real-time PCR (qRT-PCR) analysis of relative mRNA expression levels of *PCNA* and *Ki67* in the control and ATRA-treated groups. **(c)** Representative images of 5-ethynyl-2′-deoxyuridine (EdU) staining of SHF-DPCs treated with different concentrations of ATRA(0, 10^−8^, 10^−6^, and 10^−4^ M)for 24 h (scale bar = 200 μm). **(d)** Representative images of terminal deoxynucleotidyl transferase–mediated dUTP nick end labeling (TUNEL) staining of SHF-DPCs treated with different concentrations of ATRA(0, 10^−8^, 10^−6^, and 10^−4^ M) for 24 h (scale bar = 200 μm). **(e)** Flow cytometric analysis of cell cycle distribution of SHF-DPCs in the control and ATRA-treated groups (10^−4^ M) with corresponding quantitative analysis of cell proportions in each phase. **(f)** Western blot analysis of Bcl-2 and Bax expression in the control and ATRA-treated groups (10^−4^ M) with corresponding quantitative analysis. Data are presented as the mean ± standard deviation (SD). *p < 0.05; **p < 0.01; ***p < 0.001; ****p < 0.0001.

To evaluate the effects of ATRA on cashmere goat SHF-DPCs, cell viability and status were monitored using the CCK-8, EdU, and TUNEL assays. ATRA inhibited SHF-DPC viability in a concentration-dependent manner, with a critical concentration of 10^−4^ M (Figure 2a). Consistently, EdU and TUNEL assays confirmed these findings: the proportion of EdU-positive (proliferating) cells decreased significantly with increasing ATRA concentrations, and almost no EdU-positive cells were observed at 10^−4^ M (Figure 2c). In contrast, the proportion of TUNEL-positive (apoptotic) cells increased markedly at higher ATRA concentrations (10^−4^ M), whereas few TUNEL-positive cells were detected at lower concentrations (10^−8^ M; Figure 2d). These results suggest that ATRA modulates the biological behavior of cashmere goat SHF-DPCs in a dose-dependent manner.

Flow cytometric analysis was conducted to examine the effect of ATRA on the cell cycle progression of SHF-DPCs. qRT-PCR was used to measure mRNA levels of the proliferation markers *Ki67* and *PCNA*, and western blotting was employed to evaluate protein levels of the pro-apoptotic Bax and anti-apoptotic Bcl-2. Compared with the control group, SHF-DPCs treated with 10^−4^ M ATRA showed a significant increase in the proportion of cells in the G1 phase and a corresponding decrease in the proportion of cells in the S phase (Figure 2e). Furthermore, ATRA treatment significantly downregulated *Ki67* and *PCNA* expression (Figure 2b) and decreased Bcl-2 protein levels while increasing Bax levels (Figure 2f). Collectively, these findings demonstrate that ATRA inhibits proliferation and promotes apoptosis in cashmere goat SHF-DPCs.

### 3.3 Effects of ATRA on the transcriptome of cashmere goat SHF-DPCs

**Figure 3:**
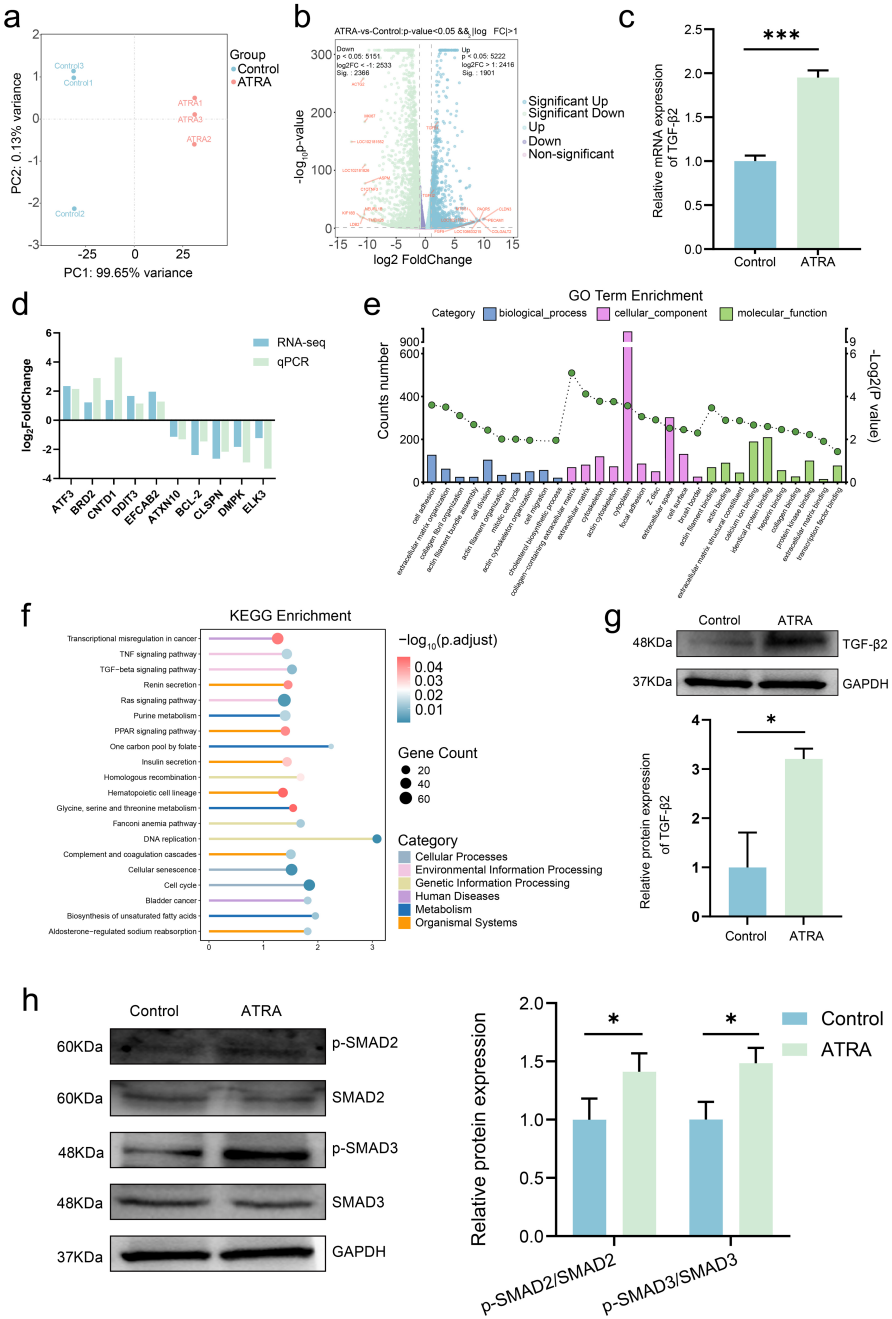
ATRA alters the transcriptome of cashmere goat SHF-DPCs. **(a)** Principal component analysis (PCA) of transcriptome sequencing data from the control and ATRA-treated (10^−4^ M, 24 h) groups. **(b)** Volcano plot showing differentially expressed genes (DEGs) between the control and ATRA-treated groups (*p* < 0.05 and |log₂ fold change (FC)| > 1). **(c)** Relative mRNA expression of transforming growth factor-β2 (TGF-*β*2) in the control and ATRA-treated groups (qRT-PCR). **(d)** Correlation between RNA sequencing and qRT-PCR data for selected DEGs based on log₂FC values; **(e)** Gene Ontology enrichment analysis of DEGs. **(f)** Kyoto Encyclopedia of Genes and Genomes (KEGG) pathway enrichment bubble plot of DEGs. **(g)** Western blot analysis of TGF-*β*2 expression in the control and ATRA-treated groups with corresponding quantitative analysis. **(h)** Western blot analysis of Smad2/3 phosphorylation levels in the control and ATRA-treated groups with corresponding quantitative analysis. Data are presented as the mean ± SD. *p < 0.05; **p < 0.01; ***p < 0.001.

To investigate the molecular mechanisms underlying ATRA-mediated regulation of SHF-DPCs, transcriptome sequencing (RNA-seq) was performed on SHF-DPCs treated with 10^−4^ M ATRA and on control cells, with three biological replicates per group. After quality control and alignment, a total of 15,944 genes were identified. Principal component analysis showed clear separation between the two groups and strong intra-group reproducibility, confirming the reliability of the sequencing data (Figure 3a). Using thresholds of |log₂FC| > 1 and *p* < 0.05, 1,901 significantly upregulated and 2,366 significantly downregulated genes were identified in the ATRA-treated group compared with the control group (Figure 3b). Notably, downregulated genes included *ASPM* (associated with cell-cycle arrest and reduced proliferation) and *MKI67* (a cell proliferation marker), consistent with our previous findings. Upregulated genes included *TGFβ1 and TGFβ2* (regulators of proliferation and apoptosis) and *FGF9* (a modulator of proliferation and apoptosis) (41–44). To validate the RNA-seq data, qRT-PCR was performed on five randomly selected DEGs. The log₂FC values of these genes obtained from qRT-PCR were consistent with those from RNA-seq, supporting the reliability of the sequencing results (Figure 3d).

GO enrichment analysis categorized DEGs into three functional ontologies: biological process (BP), cellular component (CC), and molecular function (MF). Enriched BP terms included “cell migration,” “mitotic cell cycle,” and “cell division,” all of which are closely related to our previous findings. The MF term “transcription factor binding” was also enriched, suggesting that ATRA alters transcription factor binding in SHF-DPCs (Figure 3e). Collectively, these results suggest that ATRA modulates BPs such as cell proliferation, cell cycle progression, and metabolic regulation in SHF-DPCs.

KEGG pathway enrichment analysis classified the enriched pathways into six categories: Cellular Processes, Environmental Information Processing, Human Diseases, Metabolism, Genetic Information Processing, and Organismal Systems (Figure 3f). Notable pathways included the TNF signaling pathway, which inhibits NF-κB activity and enhances TNF-mediated apoptosis (45), DNA replication, and the cell cycle—all consistent with ATRA’s effects on SHF-DPCs. However, these pathways are broadly regulatory, affecting all proliferating cells. The Ras signaling pathway was weakly regulated by ATRA, with downregulation of downstream effectors observed only in specific cell types (46). Although multiple pathways were enriched, the TGF-*β* pathway—and specifically TGF-*β*2—was prioritized for further investigation due to its robust transcriptional induction, established role in hair follicle regression and proliferation inhibition, and its strong concordance with the observed cellular phenotypes. qRT-PCR analysis confirmed that *TGFβ2* mRNA expression was significantly upregulated in the ATRA-treated group, consistent with RNA-seq results (Figure 3c). Western blotting further showed that ATRA treatment significantly increased TGF-*β*2 and Smad2/3 phosphorylation levels (pathway activation markers; Figures 3g,h). Collectively, the transcriptome sequencing data indicate that ATRA regulates cashmere goat SHF-DPCs via multiple signaling pathways, including the TGF-*β* signaling pathway, providing insights into the underlying molecular mechanisms.

### 3.4 ATRA regulates proliferation and apoptosis in cashmere goat SHF-DPCs via the TGF-*β*2/Smad2/3 pathway

**Figure 4:**
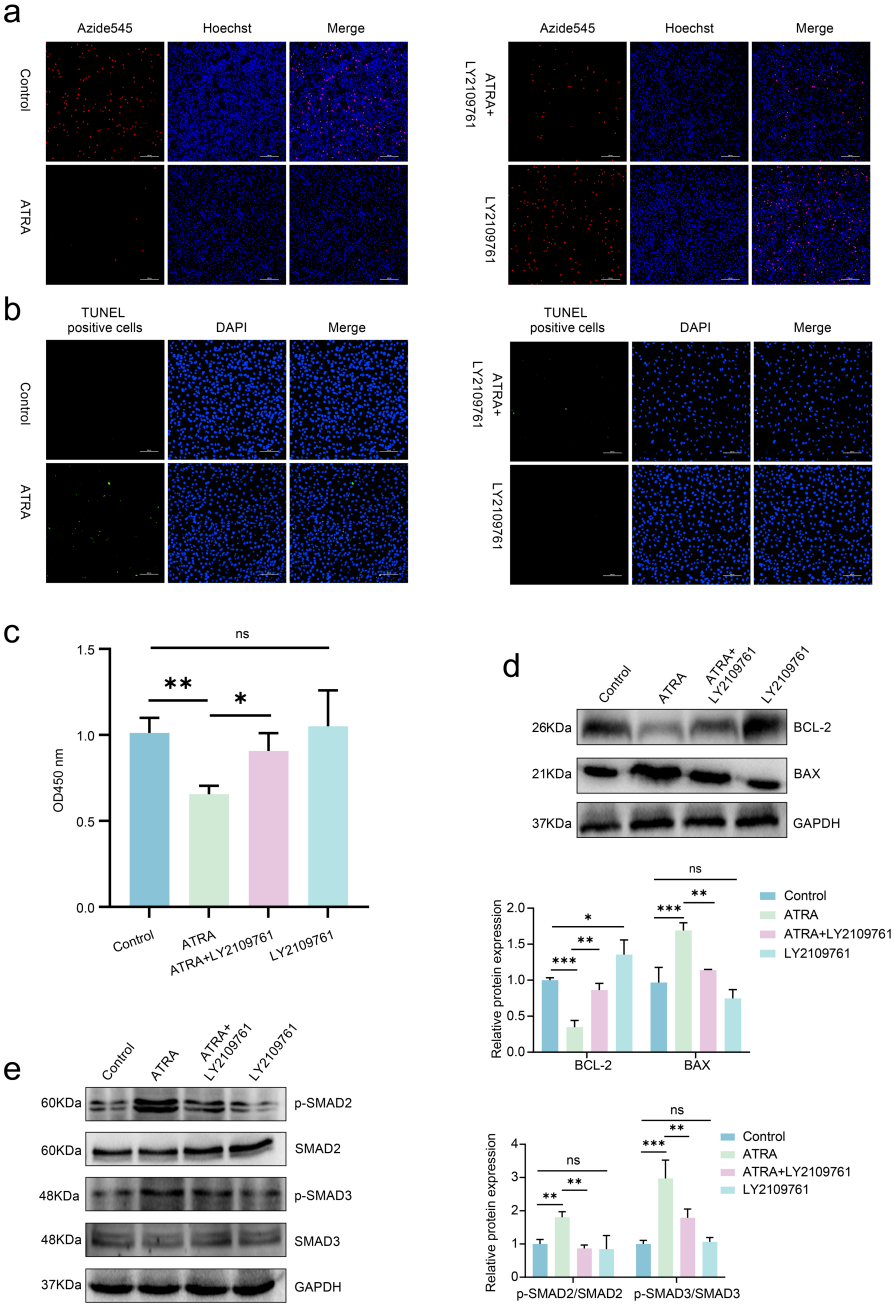
ATRA regulates proliferation and apoptosis in cashmere goat SHF-DPCs via the TGF-*β*2/Smad2/3 pathway. **(a)** Representative images of EdU staining of SHF-DPCs treated with ATRA (10^−4^ M) LY2109761 (2 μM), or ATRA + LY2109761 (scale bar = 200 μm). **(b)** Representative images of TUNEL staining of SHF-DPCs treated with ATRA, LY2109761, or ATRA + LY2109761 (scale bar = 200 μm). **(c)** CCK-8 assay to assess cell viability of SHF-DPCs treated with ATRA, LY2109761, or ATRA + LY2109761. **(d)** Western blot analysis of Bcl-2 and Bax expression in SHF-DPCs treated with ATRA, LY2109761, or ATRA + LY2109761 with corresponding quantitative analysis. **(e)** Western blot analysis of Smad2/3 phosphorylation levels in SHF-DPCs treated with ATRA, LY2109761, or ATRA + LY2109761 with corresponding quantitative analysis. Data are presented as the mean ± SD. *p < 0.05; **p < 0.01; ***p < 0.001; ns, not significant.

To further investigate whether ATRA regulates proliferation and apoptosis in SHF-DPCs via the TGF-*β*2/Smad2/3 pathway, the cells were treated with ATRA (10^−4^ M) alone, LY2109761 (2 μM; a TGF-*β* type I/II receptor inhibitor) alone, or a combination of both. The CCK-8, EdU, and TUNEL assays were used to evaluate cell proliferation and apoptosis. The ATRA-treated group showed significantly reduced cell viability compared with that in the control group, whereas the ATRA + LY2109761 group exhibited a marked recovery in viability. The LY2109761 group also showed a slight increase in viability compared with that in the control group (Figure 4c). Consistent with these results, the proportion of EdU-positive cells was significantly lower in the ATRA group than that in the control group, but this decrease was partially reversed in the ATRA + LY2109761 group. The LY2109761 group also showed a modest increase in EdU-positive cells (Figure 4a). TUNEL assays demonstrated that LY2109761 effectively mitigated ATRA-induced apoptosis (Figure 4b).

Western blot analysis was conducted to measure Bcl-2 and Bax protein levels. Compared with the ATRA alone group, the ATRA + LY2109761 group showed significantly upregulated Bcl-2 expression and downregulated Bax expression, indicating that LY2109761 attenuates ATRA-induced apoptosis and promotes cell proliferation (Figure 4d). Given that ATRA enhances Smad2/3 phosphorylation, we examined whether Smad2/3 activation is associated with ATRA-mediated regulation of SHF-DPC proliferation and apoptosis. Co-treatment with ATRA and LY2109761 reduced ATRA-induced Smad2/3 phosphorylation, with no significant difference compared with the control group (Figure 4e). Collectively, these results demonstrate that ATRA inhibits proliferation and promotes apoptosis of cashmere goat SHF-DPCs via the TGF-*β*2/Smad2/3 pathway.

## 4 Discussion

ATRA is known to play a critical role in hair follicle morphogenesis and cycle regulation (47); however, the effects of exogenously administered ATRA remain controversial. In mouse embryos lacking ATRA-degrading enzymes, excessive ATRA accumulation arrests hair follicle development at the hair germ stage—a phenotype that can be reversed by transplanting the affected skin into wild-type mice (30). Elevated ATRA also disrupts DPC-related signaling, inducing the transformation of mouse skin hair follicles into glandular structures and impairing follicular morphogenesis (48). In *in vitro* culture systems, the effective bioavailability of all-trans retinoic acid (ATRA) is markedly lower than that *in vivo*, owing to its susceptibility to photodegradation, nonspecific binding to serum components, and rapid catabolism mediated by intracellular enzymes such as CYP26 (49–51). In an in vitro study of mink hair follicle dermal papilla cells, researchers reported that exposure to all-trans retinoic acid (ATRA) at 10^−4^ M resulted in inhibitory effects (52). As human hair follicle morphogenesis and cycle regulation share similar mechanisms (1, 53), most studies have reported adverse effects of ATRA on hair growth. Nevertheless, conflicting findings exist: in vitro studies have shown that ATRA promotes cell cycle progression and enhances the viability of mouse dermal cells, exerting a positive effect on hair growth (33). Another study demonstrated that ATRA shortens the telogen phase and promotes hair growth in mice (32). ATRA has also shown beneficial effects in the treatment of human alopecia (34, 54).

Derived from specialized fibroblasts in the dermal mesenchyme, DPCs form functional fibroblast populations through epithelial–mesenchymal interactions during the initiation of hair follicle growth (55). By secreting various growth factors, DPCs regulate the proliferation of hair follicle keratinocytes during morphogenesis, thereby playing a key role in both embryonic follicle formation and postnatal follicle cycling (56, 57). In this study, SHF-DPCs from cashmere goats were used as a model to systematically investigate the effects of ATRA on SHF-DPC proliferation and apoptosis, as well as the underlying molecular mechanisms, using cell function assays, RNA-seq, and pathway rescue experiments. Our results indicate that ATRA inhibits proliferation and promotes apoptosis of cashmere goat SHF-DPCs, likely via the TGF-*β*2/Smad2/3 signaling pathway.

Consistent with previous studies (52), ATRA inhibited SHF-DPC proliferation and induced apoptosis in a concentration-dependent manner. Furthermore, ATRA treatment resulted in G1-phase cell cycle arrest, accompanied by downregulation of the anti-apoptotic protein Bcl-2 and upregulation of the pro-apoptotic protein Bax. Given that Bcl-2 and Bax act cooperatively to maintain the dynamic balance of the hair follicle cycle (58), and G1-phase arrest is associated with DNA damage responses, consistent with cell cycle checkpoint regulation (59), these findings suggest that ATRA may contribute to the induction of senescence-like features in SHF-DPCs. Notably, the threshold concentration of ATRA required to elicit responses in cashmere goat SHF-DPCs (10^−4^ M) was significantly higher than that in mink hair follicle DPCs (10^−5^ M) (53). We hypothesize that this interspecies difference arises from higher basal expression of the ATRA-metabolizing enzyme CYP26A1 in cashmere goat skin. CYP26A1 catalyzes ATRA degradation through hydroxylation, thereby reducing intracellular active ATRA levels and necessitating higher exogenous ATRA concentrations to achieve the regulatory threshold—a hypothesis supported by previous findings (60).

The apparently dual effects of ATRA on hair growth raise important questions regarding the underlying mechanisms. Accumulating evidence indicates that TGF-*β* signaling can exert both pro-apoptotic and anti-apoptotic effects depending on the cellular context. During the telogen-to-anagen transition, TGF-*β*2 activates the Smad2/3 pathway to counteract BMP-mediated inhibition, thereby promoting the shift to the anagen phase (61). Conversely, TGF-*β*2 activates the MAPK pathway to induce apoptosis of hair follicle epithelial cells, thereby inhibiting anagen progression (62–64).

RNA-seq of ATRA-treated SHF-DPCs identified 4,267 DEGs, including a significant upregulation of *TGFβ2* (log₂FC = 2.3, *p* < 0.01)—a key molecule in the TGF-*β* signaling pathway. KEGG enrichment analysis confirmed that the TGF-*β* signaling pathway was among the most significantly enriched pathways. GO functional annotation further showed that DEGs were enriched in BP terms related to proliferation and apoptosis, such as “cell migration,” “mitotic cell cycle,” and “cell division,” consistent with our experimental results. These findings suggest that ATRA modulates SHF-DPC cell cycle progression and survival via the TGF-*β* pathway. Our results showed that ATRA upregulated TGF-*β*2 protein expression and significantly increased Smad2/3 phosphorylation, consistent with the established role of the TGF-*β* pathway in hair follicle development (65). Activation of the TGF-*β*/Smad pathway is a key molecular event initiating the catagen phase (62, 66). As a core transcription factor in this pathway, Smad3 directly binds to the promoters of cell cycle–related genes to maintain cell cycle arrest (67). Transcriptomic analysis showed that among the TGF-*β* isoforms, only TGF-*β*1 and TGF-*β*2 were significantly upregulated at the transcriptional level. However, previous studies have demonstrated that TGF-*β*2 exhibits more pronounced stage-specific expression during the hair follicle regression (catagen) phase and functions as a key signaling mediator of hair follicle regression, whereas TGF-*β*1 is more broadly associated with general growth-inhibitory or fibrosis-related regulatory processes (31, 68). Therefore, TGF-*β*2 was selected for subsequent mechanistic experiments. To confirm the role of the TGF-*β*2/Smad2/3 pathway in ATRA-mediated regulation, we performed rescue experiments using LY2109761, a specific inhibitor of TGF-*β* type I/II receptors. Our findings align with those of previous studies (69, 70): LY2109761 reversed the ATRA-induced effects on SHF-DPCs. Additionally, LY2109761 treatment alone slightly increased cell viability and the proportion of EdU-positive cells relative to the control group, suggesting that the TGF-*β*2/Smad2/3 pathway maintains basal activity under physiological conditions to moderately inhibit SHF-DPC proliferation, thereby preventing excessive activation and supporting the homeostatic regulation of the hair follicle cycle (6). Collectively, our findings demonstrate that ATRA inhibits proliferation and promotes apoptosis of cashmere goat SHF-DPCs via the TGF-*β*2/Smad2/3 pathway.

Although dermal papilla cells play a pivotal role in regulating the hair follicle cycle, the effects of all-trans retinoic acid (ATRA) at the organismal level are additionally influenced by epithelial–mesenchymal interactions and systemic factors. Therefore, the findings of this study primarily reflect the cell-intrinsic regulatory mechanisms of ATRA under *in vitro* conditions and provide a reference for future *in vivo* investigations.

## 5 Conclusion

In summary, this study demonstrates that ATRA inhibits proliferation and promotes apoptosis of SHF-DPCs from Albas cashmere goats and that this regulatory effect is mediated through the TGF-*β*2/Smad2/3 signaling pathway. These findings provide a theoretical basis for developing molecular breeding strategies to improve cashmere quality and for designing potential interventions for hair follicle–related disorders.

## Data Availability

The RNA-seq data generated in this study have been deposited in the NCBI Gene Expression Omnibus (GEO) database under accession number GSE324826.

## Footnotes

1 http://www.blast2go.com/b2ghome; http://geneontology.org/

## Glossary

*α*-SMA: alpha-smooth muscle actin
ATRA: all-trans retinoic acid
ANOVA: analysis of variance
BP: biological process
CC: cellular component
CCK-8: Cell Counting Kit-8
DAPI: 4′,6-diamidino-2-phenylindole
DEGs: differentially expressed genes
DMEM: Dulbecco’s modified Eagle medium
EdU: 5-ethynyl-2′-deoxyuridine
FBS: fetal bovine serum
FC: fold change
GO: Gene Ontology
KEGG: Kyoto Encyclopedia of Genes and Genomes
PBS: phosphate-buffered saline
PHFs: primary hair follicles
qRT-PCR: quantitative real-time PCR
SHFs: secondary hair follicles
SHF-DPCs: secondary hair follicle–derived dermal papilla cells
SD: standard deviation
TGF-*β*: transforming growth factor beta
TUNEL: terminal deoxynucleotidyl transferase dUTP nick end labeling

## References

[1] Millar SE. Molecular mechanisms regulating hair follicle development. J Invest Dermatol. (2002) 118:216–25. doi: 10.1046/j.0022-202x.2001.01670.x, 11841536

[2] Potter CS Kern MJ Baybo MA Pruett ND Godwin AR Sundberg JP . Dysregulated expression of sterol O-acyltransferase 1 (Soat1) in the hair shaft of Hoxc13 null mice. Exp Mol Pathol. (2015) 99:441–4. doi: 10.1016/j.yexmp.2015.08.016, 26321246 PMC4679597

[3] Stenn KS Paus R. Controls of hair follicle cycling. Physiol Rev. (2001) 81:449–94. doi: 10.1152/physrev.2001.81.1.449, 11152763

[4] Diao X Yao L Wang X Li S Qin J Yang L . Hair follicle development and cashmere traits in albas goat kids. Animals. (2023) 13:617. doi: 10.3390/ani13040617, 36830404 PMC9951752

[5] Rile N Liu Z Gao L Qi J Zhao M Xie Y . Expression of vimentin in hair follicle growth cycle of inner Mongolian cashmere goats. BMC Genomics. (2018) 19:38. doi: 10.1186/s12864-017-4418-7, 29320989 PMC5764018

[6] Paus R Cotsarelis G. The biology of hair follicles. N Engl J Med. (1999) 341:491–7. doi: 10.1056/NEJM199908123410706, 10441606

[7] Welle MM. Basic principles of hair follicle structure, morphogenesis, and regeneration. Vet Pathol. (2023) 60:732–47. doi: 10.1177/03009858231176561, 37272599

[8] Duan C Zhang L Gao K Guo Y Liu Y Zhang Y. Cashmere production, skin characteristics, and mutated genes in crimped cashmere fibre goats. Animal. (2022) 16:100565. doi: 10.1016/j.animal.2022.100565, 35714387

[9] Gong G Fan Y Li W Yan X Yan X Zhang L . Identification of the key genes associated with different hair types in the Inner mongolia cashmere goat. Animals. (2022) 12:1456. doi: 10.3390/ani12111456 35681921 PMC9179306

[10] Harland DP Plowman JE. Development of hair fibres. Adv Exp Med Biol. (2018) 1054:109–54. doi: 10.1007/978-981-10-8195-8_10, 29797272

[11] Driskell RR Clavel C Rendl M Watt FM. Hair follicle dermal papilla cells at a glance. J Cell Sci. (2011) 124:1179–82. doi: 10.1242/jcs.082446 21444748 PMC3115771

[12] Zhang HL Qiu XX Liao XH. Dermal papilla cells: from basic research to translational applications. Biology. (2024) 13:842. doi: 10.3390/biology13100842, 39452150 PMC11504027

[13] Paus R Langan EA Vidali S Ramot Y Andersen B. Neuroendocrinology of the hair follicle: principles and clinical perspectives. Trends Mol Med. (2014) 20:559–70. doi: 10.1016/j.molmed.2014.06.002, 25066729

[14] Soma T Hibino T. Dominant bcl-2 expression during telogen–anagen transition phase in human hair. J Dermatol Sci. (2004) 36:183–5. doi: 10.1016/j.jdermsci.2004.09.003, 15541642

[15] Fuchs E. Scratching the surface of skin development. Nature. (2007) 445:834–42. doi: 10.1038/nature05659, 17314969 PMC2405926

[16] Rendl M Polak L Fuchs E. BMP signaling in dermal papilla cells is required for their hair follicle-inductive properties. Genes Dev. (2008) 22:543–57. doi: 10.1101/gad.1614408, 18281466 PMC2238674

[17] Badouel C McNeill H. SnapShot: the hippo signaling pathway. Cell. (2011) 145:484–484.e1. doi: 10.1016/j.cell.2011.04.009, 21529719

[18] Lin HY Yang LT. Differential response of epithelial stem cell populations in hair follicles to TGF-β signaling. Dev Biol. (2013) 373:394–406. doi: 10.1016/j.ydbio.2012.10.021, 23103542

[19] Echeverría C Montorfano I Tapia P Riedel C Cabello-Verrugio C Simon F. Endotoxin-induced endothelial fibrosis is dependent on expression of transforming growth factors β1 and β2. Infect Immun. (2014) 82:3678–86. doi: 10.1128/iai.02158-14, 24935972 PMC4187819

[20] Wu J Fu R Liu Z Li G Huang X Xue Y . Cell proliferation downregulated by TGF-*β*2-triggered G1/S checkpoint in clinical CAFs. Cell Cycle. (2017) 16:172–8. doi: 10.1080/15384101.2016.1253641, 27880067 PMC5283810

[21] Yao K Ye PP Tan J Tang XJ Shen Tu XC. Involvement of PI3K/akt pathway in TGF-beta2-mediated epithelial mesenchymal transition in human lens epithelial cells. Ophthalmic Res. (2008) 40:69–76. doi: 10.1159/000113884, 18223299

[22] Shin HS Park SY Hwang ES Lee D-G Mavlonov GT Yi T-H. Ginsenoside F2 reduces hair loss by controlling apoptosis through the sterol regulatory element-binding protein cleavage activating protein and transforming growth factor-β pathways in a dihydrotestosterone-induced mouse model. Biol Pharm Bull. (2014) 37:755–63. doi: 10.1248/bpb.b13-00771, 24789999

[23] Su R Fan Y Qiao X Li X Zhang L Li C . Transcriptomic analysis reveals critical genes for the hair follicle of Inner Mongolia cashmere goat from catagen to telogen. PLoS One. (2018) 13:e0204404. doi: 10.1371/journal.pone.0204404, 30356261 PMC6200190

[24] Liang C Qiao G Liu Y Tian L Hui N Li J . Overview of all-trans-retinoic acid (ATRA) and its analogues: structures, activities, and mechanisms in acute promyelocytic leukaemia. Eur J Med Chem. (2021) 220:113451. doi: 10.1016/j.ejmech.2021.113451, 33895500

[25] Szymański Ł Skopek R Palusińska M Schenk T Stengel S Lewicki S . Retinoic acid and its derivatives in skin. Cells. (2020) 9:2660. doi: 10.3390/cells9122660 33322246 PMC7764495

[26] Liu Y Wen Q Chen XL Yang SJ Gao L Gao L . All-trans retinoic acid arrests cell cycle in leukemic bone marrow stromal cells by increasing intercellular communication through connexin 43-mediated gap junction. J Hematol Oncol. (2015) 8:110. doi: 10.1186/s13045-015-0212-7 26446715 PMC4597383

[27] Manolescu DC Jankowski M Danalache BA Wang D Broderick TL Chiasson J-L . All-trans retinoic acid stimulates gene expression of the cardioprotective natriuretic peptide system and prevents fibrosis and apoptosis in cardiomyocytes of obese Ob/Ob mice. Appl. Physiol. Nutr. Metab. (2014) 39:1127–36. doi: 10.1139/apnm-2014-0005 25017112

[28] Uniyal S Dhasmana A Tyagi A Muyal JP. ATRA reduces inflammation and improves alveolar epithelium regeneration in emphysematous rat lung. Biomed Pharmacother. (2018) 108:1435–50. doi: 10.1016/j.biopha.2018.09.166, 30372846

[29] Kane MA. "Retinoic acid homeostasis and disease". In: Current Topics in Developmental Biology, vol. 161. Cambridge, MA: Academic Press. (2025). p. 201–33. 39870434 10.1016/bs.ctdb.2024.11.001

[30] Okano J Lichti U Mamiya S Aronova M Zhang G Yuspa SH . Increased retinoic acid levels through ablation of Cyp26b1 determine the processes of embryonic skin barrier formation and peridermal development. J Cell Sci. (2012) 125:1827–36. doi: 10.1242/jcs.101550, 22366455 PMC3346831

[31] Foitzik K Spexard T Nakamura M Halsner U Paus R. Towards dissecting the pathogenesis of retinoid-induced hair loss: all-trans retinoic acid induces premature hair follicle regression (catagen) by upregulation of transforming growth factor-beta2 in the dermal papilla. J Invest Dermatol. (2005) 124:1119–26. doi: 10.1111/j.0022-202X.2005.23686.x, 15955085

[32] Bazzano G Terezakis N Attia H Bazzano A Dover R Fenton D . Effect of retinoids on follicular cells. J Invest Dermatol. (1993) 101:138S–42S. doi: 10.1111/1523-1747.ep12363264, 8392099

[33] Zheng L Du Y Zhang L Jin F Li W Zhou X . Enhanced therapeutic effects of all-trans retinoic acid nanostructured lipid carrier composite gel drug delivery system for alopecia areata. J Nanobiotechnol. (2025) 23:351. doi: 10.1186/s12951-025-03407-w, 40380336 PMC12083027

[34] Kwon OS Pyo HK Oh YJ Han JH Lee SR Chung JH . Promotive effect of minoxidil combined with all-trans retinoic acid (tretinoin) on human hair growth in vitro. J Korean Med Sci. (2007) 22:283–9. doi: 10.3346/jkms.2007.22.2.283, 17449938 PMC2693596

[35] Liu X Zhang H Gao L Yin Y Pan X Li Z . Negative interplay of retinoic acid and TGF-β signaling mediated by TG-interacting factor to modulate mouse embryonic palate mesenchymal-cell proliferation. Birth Defects Res B Dev Reprod Toxicol. (2014) 101:403–9. doi: 10.1002/bdrb.21130, 25477235

[36] Sarper M Cortes E Lieberthal TJ del Río Hernández A. ATRA modulates mechanical activation of TGF-β by pancreatic stellate cells. Sci Rep. (2016) 6:27639. doi: 10.1038/srep27639 27375161 PMC4931506

[37] Namachivayam K MohanKumar K Arbach D Jagadeeswaran R Jain SK Natarajan V . All-trans retinoic acid induces TGF-β2 in intestinal epithelial cells via RhoA- and p38α MAPK-mediated activation of the transcription factor ATF2. PLoS One. (2015) 10:e0134003. doi: 10.1371/journal.pone.0134003, 26225425 PMC4520553

[38] Zhu B Xu T Yuan J Guo X Liu D. Transcriptome sequencing reveals differences between primary and secondary hair follicle-derived dermal papilla cells of the cashmere goat (*capra hircus*). PLoS One. (2013) 8:e76282. doi: 10.1371/journal.pone.0076282, 24069460 PMC3777969

[39] Gong W Liu J Mu Q Chahaer T Liu J Ding W . Melatonin promotes proliferation of Inner mongolia cashmere goat hair follicle papilla cells through Wnt10b. Genomics. (2024) 116:110844. doi: 10.1016/j.ygeno.2024.110844, 38608737

[40] He X Chao Y Zhou G Chen Y. Fibroblast growth factor 5-short (FGF5s) inhibits the activity of FGF5 in primary and secondary hair follicle dermal papilla cells of cashmere goats. Gene. (2016) 575:393–8. doi: 10.1016/j.gene.2015.09.034, 26390813

[41] Bikeye SNN Colin C Marie Y Vampouille R Ravassard P Rousseau A . ASPM-associated stem cell proliferation is involved in malignant progression of gliomas and constitutes an attractive therapeutic target. Cancer Cell Int. (2010) 10:1. doi: 10.1186/1475-2867-10-1 20142996 PMC2817685

[42] Zhang Y Alexander PB Wang XF. TGF-β family signaling in the control of cell proliferation and survival. Cold Spring Harb Perspect Biol. (2017) 9:a022145. doi: 10.1101/cshperspect.a022145, 27920038 PMC5378054

[43] Yang F Whelan EC Guan X Deng B Wang S Sun J . FGF9 promotes mouse spermatogonial stem cell proliferation mediated by p38 MAPK signalling. Cell Prolif. (2021) 54:e12933. doi: 10.1111/cpr.12933, 33107118 PMC7791179

[44] Jia Q Zhang S Wang D Liu J Luo X Liu Y . Regulatory effects of FGF9 on dermal papilla cell proliferation in small-tailed han sheep. Genes. (2023) 14:1106. doi: 10.3390/genes14051106 37239467 PMC10218283

[45] Chen T Zhang X Zhu G Liu H Chen J Wang Y . Quercetin inhibits TNF-α induced HUVECs apoptosis and inflammation via downregulating NF-kB and AP-1 signaling pathway in vitro. Medicine (Baltimore). (2020) 99:e22241. doi: 10.1097/MD.0000000000022241, 32957369 PMC7505396

[46] Wang AG Song YN Chen J Li HL Dong JY Cui HP . Activation of RAS/ERK alone is insufficient to inhibit RXRα function and deplete retinoic acid in hepatocytes. Biochem Biophys Res Commun. (2014) 452:801–7. doi: 10.1016/j.bbrc.2014.09.007, 25218146

[47] Everts HB. Endogenous retinoids in the hair follicle and sebaceous gland. Biochim Biophys Acta. (2012) 1821:222–9. doi: 10.1016/j.bbalip.2011.08.017, 21914489 PMC3237781

[48] Viallet JP Ruberte E du Manoir S Krust A Zelent A Dhouailly D. Retinoic acid-induced glandular metaplasia in mouse skin is linked to the dermal expression of retinoic acid receptor beta mRNA. Dev Biol. (1991) 144:424–8. doi: 10.1016/0012-1606(91)90434-5, 1849102

[49] Napoli JL. Physiological insights into all-trans-retinoic acid biosynthesis. Biochim Biophys Acta. (2012) 1821:152–67. doi: 10.1016/j.bbalip.2011.05.004, 21621639 PMC3179567

[50] Duester G. Retinoic acid synthesis and signaling during early organogenesis. Cell. (2008) 134:921–31. doi: 10.1016/j.cell.2008.09.002, 18805086 PMC2632951

[51] Blomhoff R Blomhoff HK. Overview of retinoid metabolism and function. J Neurobiol. (2006) 66:606–30. doi: 10.1002/neu.20242, 16688755

[52] Nan W Li G Si H Lou Y Wang D Guo R . All-trans-retinoic acid inhibits mink hair follicle growth via inhibiting proliferation and inducing apoptosis of dermal papilla cells through TGF-β2/Smad2/3 pathway. Acta Histochem. (2020) 122:151603. doi: 10.1016/j.acthis.2020.151603, 33066831

[53] Rendl M Lewis L Fuchs E. Molecular dissection of mesenchymal-epithelial interactions in the hair follicle. PLoS Biol. (2005) 3:e331. doi: 10.1371/journal.pbio.0030331, 16162033 PMC1216328

[54] Yoo HG Chang IY Pyo HK Kang YJ Lee SH Kwon OS . The additive effects of minoxidil and retinol on human hair growth in vitro. Biol Pharm Bull. (2007) 30:21–6. doi: 10.1248/bpb.30.21, 17202653

[55] Sari ARP Rufaut NW Jones LN Sinclair RD. Characterization of ovine dermal papilla cell aggregation. Int J Trichol. (2016) 8:121–9. doi: 10.4103/0974-7753.188966 PMC5007918 27625564

[56] Hsu YC Pasolli HA Fuchs E. Dynamics between stem cells, niche, and progeny in the hair follicle. Cell. (2011) 144:92–105. doi: 10.1016/j.cell.2010.11.049, 21215372 PMC3050564

[57] Zhu B Xu T Zhang Z Ta N Gao X Hui L . Transcriptome sequencing reveals differences between anagen and telogen secondary hair follicle-derived dermal papilla cells of the cashmere goat (*capra hircus*). Physiol Genomics. (2014) 46:104–11. doi: 10.1152/physiolgenomics.00132.2013, 24326349

[58] Müller-Röver S Rossiter H Lindner G Peters EM Kupper TS Paus R. Hair follicle apoptosis and bcl-2. J Invest Dermatol Symp Proc. (1999) 4:272–7. doi: 10.1038/sj.jidsp.5640228, 10674380

[59] Gire V Dulic V. Senescence from G2 arrest, revisited. Cell Cycle (georgetown, Tex). (2015) 14:297–304. doi: 10.1080/15384101.2014.1000134, 25564883 PMC4353294

[60] Topletz AR Tripathy S Foti RS Shimshoni JA Nelson WL Isoherranen N. Induction of CYP26A1 by metabolites of retinoic acid: evidence that CYP26A1 is an important enzyme in the elimination of active retinoids. Mol Pharmacol. (2015) 87:430–41. doi: 10.1124/mol.114.096784, 25492813 PMC4352583

[61] Hsu YC Li L Fuchs E. Emerging interactions between skin stem cells and their niches. Nat Med. (2014) 20:847–56. doi: 10.1038/nm.3643, 25100530 PMC4358898

[62] Hibino T Nishiyama T. Role of TGF-beta2 in the human hair cycle. J Dermatol Sci. (2004) 35:9–18. doi: 10.1016/j.jdermsci.2003.12.003, 15194142

[63] Foitzik K Lindner G Mueller-Roever S Maurer M Botchkareva N Botchkarev V . Control of murine hair follicle regression (catagen) by TGF-beta1 in vivo. FASEB J. (2000) 14:752–60. doi: 10.1096/fasebj.14.5.752, 10744631

[64] Soma T Ogo M Suzuki J Takahashi T Hibino T. Analysis of apoptotic cell death in human hair follicles in vivo and in vitro. J Invest Dermatol. (1998) 111:948–54. doi: 10.1046/j.1523-1747.1998.00408.x, 9856801

[65] Li Z Ryu SW Lee J Choi K Kim S Choi C. Protopanaxatirol type ginsenoside re promotes cyclic growth of hair follicles via inhibiting transforming growth factor β signaling cascades. Biochem Biophys Res Commun. (2016) 470:924–9. doi: 10.1016/j.bbrc.2016.01.148, 26820528

[66] Peters EMJ Hansen MG Overall RW Peters EM Nakamura M Pertile P . Control of human hair growth by neurotrophins: brain-derived neurotrophic factor inhibits hair shaft elongation, induces catagen, and stimulates follicular transforming growth factor beta2 expression. J Invest Dermatol. (2005) 124:675–85. doi: 10.1111/j.0022-202X.2005.23648.x, 15816823

[67] Granados-Aparici S Hardy K Franks S Sharum IB Waite SL Fenwick MA. SMAD3 directly regulates cell cycle genes to maintain arrest in granulosa cells of mouse primordial follicles. Sci Rep. (2019) 9:6513. doi: 10.1038/s41598-019-42878-4, 31015579 PMC6478827

[68] Soma T Tsuji Y Hibino T. Involvement of transforming growth factor-beta2 in catagen induction during the human hair cycle. J Invest Dermatol. (2002) 118:993–7. doi: 10.1046/j.1523-1747.2002.01746.x, 12060393

[69] Zhong G Zhao Q Chen Z Yao T. TGF-β signaling promotes cervical cancer metastasis via CDR1as. Mol Cancer. (2023) 22:66. doi: 10.1186/s12943-023-01743-9, 37004067 PMC10064584

[70] Wang X Gu C Shang F Jin R Zhou J Gao Z. Inhibitory effect of the LY2109761 on the development of human keloid fibroblasts. Anal Cell Pathol (Amst). (2021) 2021:8883427. doi: 10.1155/2021/8883427 33628711 PMC7889383

